# Parry–Romberg syndrome associated with *en coup de sabre* in a patient from South Sudan – a rare entity from East Africa: a case report

**DOI:** 10.1186/s13256-019-2063-2

**Published:** 2019-05-03

**Authors:** Jimmy Girgis William Abdelnour, Youeil Girgis William Abdelnour, Rose-Mery Amin Boushra Kerollos, Ziryab Imad Taha Mahmoud

**Affiliations:** 1 Abu-Rof Clinic, Omdurman, Sudan; 2 Khartoum North Teaching Hospital, Khartoum, Sudan; 3 Haj El-Safi Teaching Hospital, Khartoum, Sudan

**Keywords:** Parry–Romberg disease, Linear scleroderma, *En coup de sabre*, Progressive hemifacial atrophy

## Abstract

**Background:**

Parry–Romberg syndrome, also known as progressive hemifacial atrophy, is a rare degenerative disorder with numerous distinctive clinical presentations. It is usually slowly progressive, occurring more in females, and affects primarily one side of the face; it causes unilateral atrophy and loss of skin, subcutaneous tissue, muscles, and bones, and can even extend to oral structures. Other involvements that can occur are ocular and neurological; however, it is frequently associated with linear scleroderma, known as *en coup de sabre*. The etiology of the disorder is unknown, although some consider it a variant of morphea (localized scleroderma) and others proposed a malfunction of the sympathetic system as a cause. Imaging studies can support diagnosis and reveal the extent of the disease. Moreover, with the wide systemic involvement in such a condition, a multidisciplinary approach is crucial.

**Case presentation:**

A 35-year-old Dinka woman presented with left hemifacial atrophy associated with left-sided body hemihypoesthesia and glaucoma with overlapping linear scleroderma “*en coup de sabre”* for 5 years.

**Conclusions:**

Parry–Romberg syndrome is a very rare entity causing progressive hemifacial atrophy that could also be associated with linear scleroderma. It has devastating outcomes due to its various systemic involvements; therefore, a multidisciplinary approach is required together with further studies to be performed in order to identify the key etiology and construct a clear guideline for management.

## Background

Parry–Romberg syndrome (PRS), also known as progressive hemifacial atrophy, was originally described by Caleb Hillier Parry in 1815, then it was described with further details by Moritz Heinrich Romberg in 1846 and in 1871 Eulenberg established the term “progressive facial hemiatrophy” [1–3]. It is a developmental craniofacial disorder of unknown etiology characterized by a slowly progressive unilateral facial atrophy and is associated with different systemic manifestations. In particular, it is associated with maxillofacial manifestations (wasting of masticatory muscles, delayed ipsilateral tooth eruption, unilateral tongue atrophy, jaw hypoplasia), and neurological (trigeminal neuralgia, migraine, seizures) and ophthalmologic abnormalities (enophthalmos, glaucoma, endothelial precipitates, Horner’s syndrome, ophthalmoplegia) [4–9]. Moreover, it is frequently associated with linear scleroderma and referred to as *en coup de sabre* [10, 11].

Onset of the disease is in childhood, typically during the first two decades of life, although later onset has previously been described. Usually, the atrophy progresses slowly for several years and afterward it becomes stable with females being affected more than males [11, 12].

Almost two centuries after this disease was defined, the etiology has still not been determined. Autoimmune disease and other causes like metabolic disorders and trauma have been postulated; sometimes, familial cases with autosomal dominant inheritance have been seen.

Concerning the treatment, there is no cure although reconstructive surgery with immunosuppressive drugs have been used and showed considerable results [10, 13].

What is unusual about the present case is the rather late onset at age of 30, the static type of progression, and the hemihypoesthesia.

## Case presentation

A 35-year-old Dinka woman from South Sudan presented with left-sided facial asymmetry associated with numbness on the left side of her body and deteriorating vision in her left eye. Her symptoms started 5 years earlier with slowly progressive left-sided facial atrophy associated with reduced vision in her left eye and early morning blurred vision. Two years later she started feeling numbness on the left side of her body (upper limb and lower limb spontaneously). Numbness and atrophy became static in the last year with further deterioration in vision.

She never had any seizures or history of a psychiatric illness. There was no family history of a similar condition; she was not on any medication or known to have any chronic illness.

**Fig. 1:**
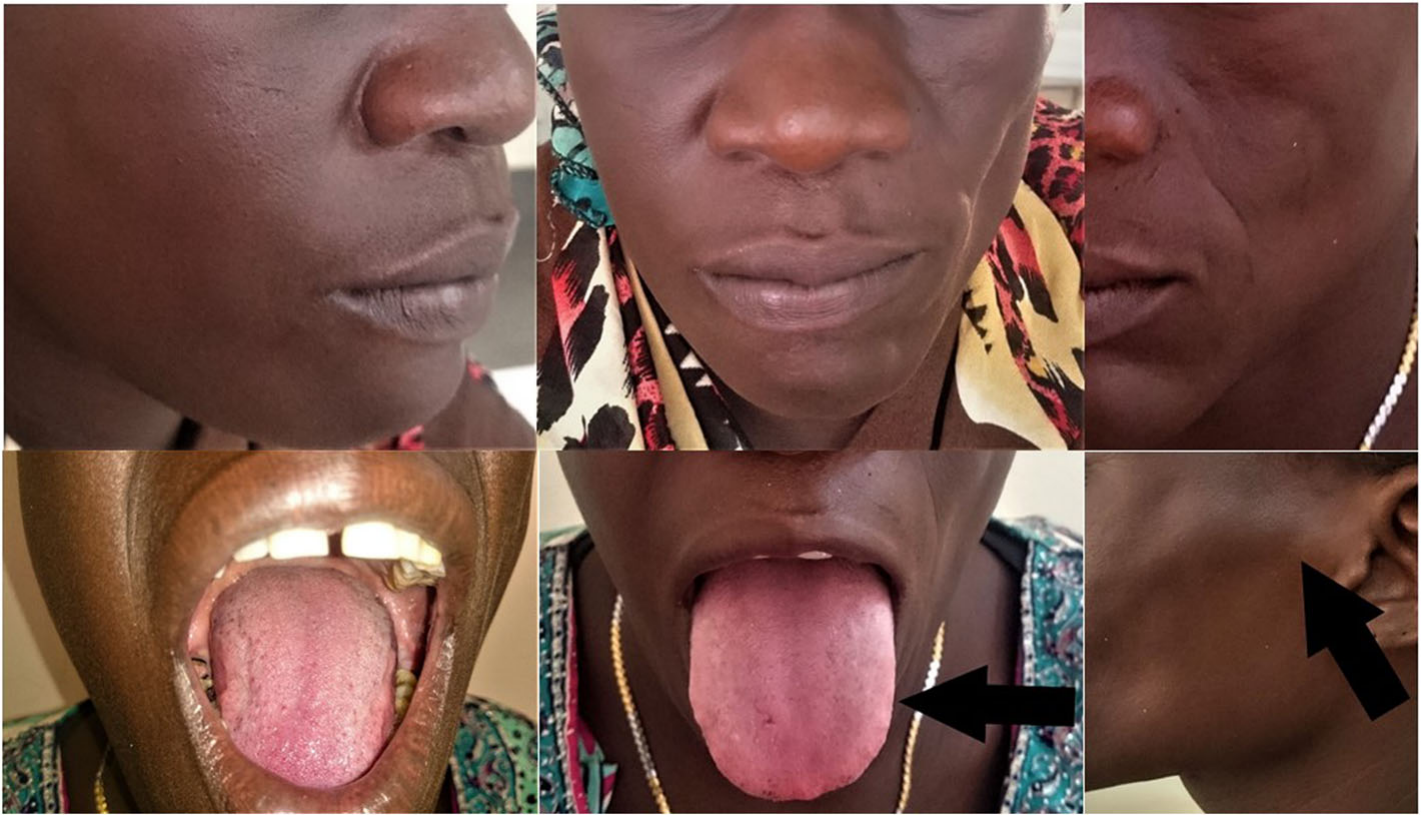
Photographs of the patient from different views showing the obvious atrophy involving the left side of her face, minimal tongue atrophy (*arrow*), clear demarcation of *en coup de sabre* (*arrow*), and the intraoral dental involvement

On examination, there was noticeable left-sided facial atrophy extending from the frontal bone to mental region associated with enophthalmos affecting her left eye, minimal tongue atrophy on the same side, and clear *coup de sabre* in her left cheek (Fig. 1). An intraoral examination revealed minimal tongue atrophy on the left side, badly decayed right maxillary third molar together with the mandibular second and third molars, and there were caries on left mandibular and maxillary third molars.

**Fig. 2:**
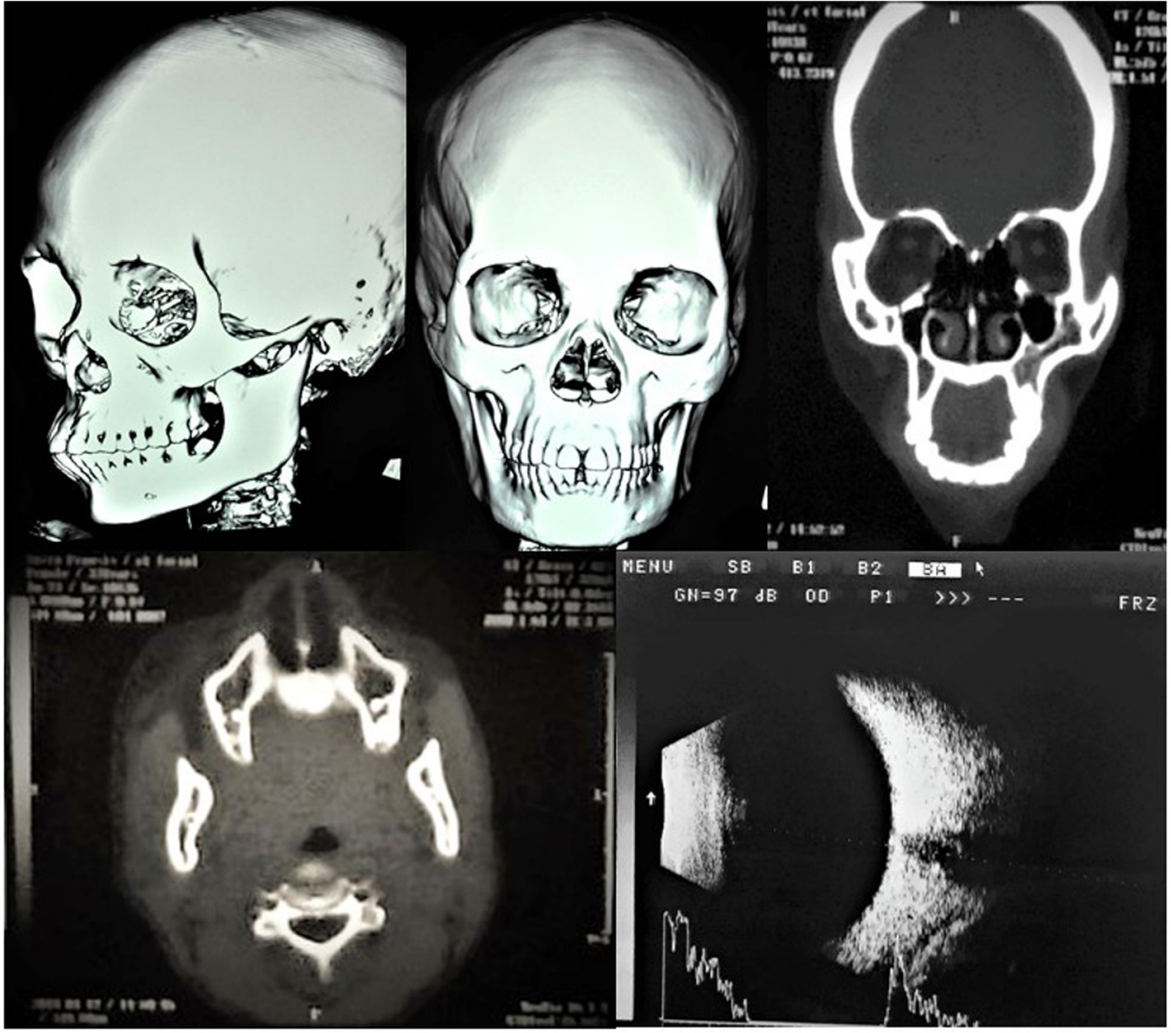
Brightness scan (ophthalmic ultrasonography) of the left eye showing no abnormalities; three-dimensional computed tomography of facial bones showing mild left zygomatic bone involvement and computed tomography showing decreased size of orbital bones on left side together with atrophy of facial subcutaneous and masseter muscle on the affected side

Her cranial nerves were intact, however, there was decreased sensation (fine touch and pin prick) affecting the left side of her body (including the left side of her face), although tone, reflexes, and proprioception were all intact. An examination of her right side was unremarkable. An ophthalmic examination showed a left eye visual acuity (VA) of 6/40, keratic precipitates, immature cataract, raised intraocular pressure (IOP) of 26, and hazy fundoscopy; however, she had a normal right eye with VA of 6/6 and IOP of 12.2. A brightness scan (B-scan) was performed and it was normal in both eyes (Fig. 2). Other systemic examinations of her heart, chest, and abdomen were unremarkable.

A diagnosis of PRS with overlapped linear scleroderma was postulated and further radiologic and laboratory assessments were made.

General routine investigations revealed normocytic normochromic anemia probably due to amoebiasis infection found in her stool sample (no other agents were seen such as ova or worms) which was corrected after treatment. Liver and renal function tests were within normal ranges as was her vitamin B_12_ level. Furthermore, rheumatoid factor was positive yet antinuclear antibody (ANA) profile and anti-cyclic citrullinated peptide (CCP) tests were all negative. Laboratory results are shown in Table 1.

**Table 1:** Lab Test Results

Laboratory Results					
CBC	*Hb*	10.6	ESR	10 mm/hour	
	*PCV*	32.1	Vitamin B_12_	412 pg/mL (221–950)	
	*RBCs*	3.99	Rh. factor	> 8 IU/mL *Positive*	
	*MCV*	80.5	Anti-CCP	9.7 AU/mL	*Negative*
	*MCH*	26.6			
	*MCHC*	33		*SM*	*Negative*
	*TWBCs*	3900		*SS-A*	*Negative*
	*N*	54%		*Ro52*	*Negative*
	*L*	37%		*SS-B*	*Negative*
	*M*	8%		*ScL 70*	*Negative*
	*E*	1%	ANA profile	*JO-1*	*Negative*
	*PLT*	265,000		*DsDNA*	*Negative*
RFT	*Urea*	24		*Nucleosomes*	*Negative*
	*Creatinine*	0.7		*Histones*	*Negative*
	*Na* ^*+*^	132		*Rib p-protein*	*Negative*
	*K* ^*+*^	3.6		*AMA M2*	*Negative*
	*Ca* ^*2+*^	9.9		*PCNA*	*Negative*
	*PO* ^*3+*^	3.4		*PM-SCL*	*Negative*
LFT	*T. protein*	*7.3*		*CENPB*	*Negative*
				*Nrnp/SM*	*Negative*
	*S. albumin*	*4.5*			
	*Globulin*	*2.8*			
	*T. bilirubin*	*0.52*			
	*D. bilirubin*	*0.3*			
	*I. bilirubin*	*0.22*			
	*ALT*	*16*			
	*AST*	*20*			
	*ALP*	*72*			

*ALP* alkaline phosphatase, *ALT* alanine aminotransferase, *AMA M2* anti-mitochondrial M2 antibody, *AST* aspartate aminotransferase, *ANA* antinuclear antibody, *CBC* complete blood count, *CCP* cyclic citrullinated peptide, *CENPB* centromere protein B, *D*. direct, *Ds* double-stranded, *E* erythrocyte, *ESR* erythrocyte sedimentation rate, *Hb* hemoglobin, *I*. indirect, *L* leukocyte, *LFT* liver function test, *M* monocyte, *MCH* mean corpuscular hemoglobin, *MCHC* mean corpuscular hemoglobin concentration, *MCV* mean corpuscular volume, *N* neutrophil, *PCNA* proliferating cell nuclear antigen, *PCV* packed cell volume, *PLT* platelet, *PM-SCL* polymyositis/scleroderma (exosome) autoantigen, *RBC* red blood cells, *RFT* renal function test, *Rh*. rhesus, *Rib* ribosomal, *S*. serum, *SS-A* Sjögren syndrome antigen A or Ro, *SS-B* Sjögren syndrome antigen B or La, *T*. total, *TWBC* total white blood cell

**Fig. 3:**
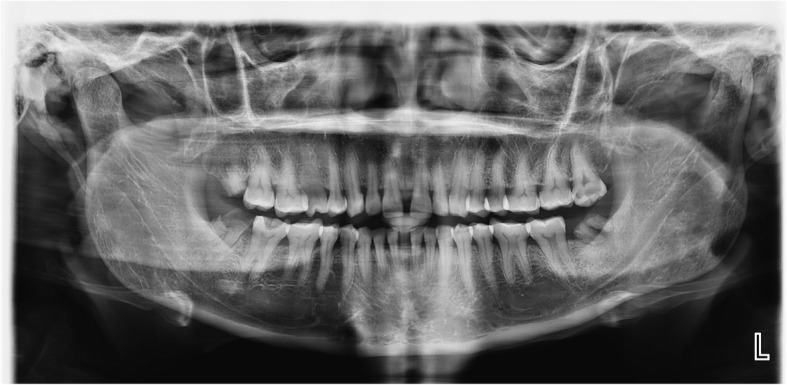
Orthopantomogram with multiple oral cavities

Radiological imaging with computed tomography (CT) scan of her facial bones revealed mild asymmetry of her face with early affection of the left zygomatic bone. There was obvious atrophy involving left-sided facial subcutaneous tissue extending to the masseter muscle (Fig. 2). Moreover, an orthopantomogram (OPG) showed multilocular radiolucency at the left angle of her mandible with remaining roots in the left mandibular third molar, decay in the maxillary left third molar (appeared as radiolucency in the enamel and dentine occlusally), an unerupted right maxillary third molar, and badly decayed mandibular third molar teeth (Fig. 3).

An electroencephalogram (EEG) revealed normal study: normal 9 Hz, α-rhythm in awake EEG with no epileptiform activity seen.

Two skin biopsies were requested that reported: skin with hyperkeratosis, follicular plugging, thin stratum Malpighii, and focal vacuolar degeneration of the basal layer with presence of dermal thick collagen fibers and mononuclear inflammatory cellular infiltration. This picture was consistent with linear scleroderma overlapping PRS.

## Discussion

PRS is a rare neurocutaneous disorder of unknown origin. It is characterized by slowly progressive facial hemiatrophy affecting all tissue layers from skin to bone and frequently (21–31%) overlaps with a condition known as linear scleroderma “*en coup de sabre*.” It has a prevalence rate of 1 in 70,000 with no difference in rates between different ethnic groups. However, only four cases were reported from Africa in the English language literature: Gueye A, *et al.* [14], Singh M, *et al.* [15], Ilhame N, *et al.* [16], and Elsaid N, *et al.* [17] from Senegal, Libya, Morocco, and Egypt, respectively. It affects mainly females and usually involves the left side of the face. In most patients (71%), disease onset commences before the age of 15 years and only 8% had an onset after the age of 25 years, as was the case in our patient. Earlier onset and longer duration of PRS have been reported to relate to increased severity of the disease [11, 12, 18–20]

A cerebral disturbance of fat metabolism and atrophic malformation of cervical sympathetic nervous system have been proposed as the primary cause. Other possible factors that are involved in the pathogenesis include trauma, infections (*Borrelia burgdorferi* and viral), heredity, endocrine disturbances, and autoimmunity [21, 22].

It is associated with different systemic manifestations. It commonly affects the dermatomes of one or multiple branches of the trigeminal nerve with atrophy of the skin and underlying structures (soft tissues, muscles, and bones) and can also affect the eye (enophthalmos, uveitis, retinal vasculitis, glaucoma, central retinal artery occlusion, heterochromic iridocyclitis, restrictive strabismus, Coats disease, papillitis, optic atrophy, and neuroretinitis), the pupil (Horner syndrome, pupillary abnormalities), and hair (band-like alopecia). Also, various oral involvements have been described (unilateral tongue atrophy, jaw hypoplasia, wasting of the muscles of mastication, short roots on affected side, deficiency of soft and hard palate, delayed ipsilateral tooth eruption, and unilateral crossbite). The most commonly seen neurological involvements are epilepsy (60.5%) followed by pain (44.2%); moreover, cerebral vascular malformations have been seen (11.5%) [4, 12].

Multiple treatment modalities have been mentioned in the literature including:

I. ***Medical***

Corticosteroids (topical and intralesional), antioxidants, immunosuppressant (methotrexate), and retinoids.

II. ***Surgical***

Fascia grafts, muscle grafts, pedicle flaps, microvascular free flaps, and free fat grafts – injection of aspirated fat and alloplastic graft materials. Some surgical modalities classify the depression type caused by the disease and afterward select the surgical technique to be used [22–24].

## Conclusion

PRS is a very rare entity causing progressive hemifacial atrophy that could also be associated with linear scleroderma.

It has devastating outcomes due to its various systemic involvements; therefore, a multidisciplinary approach is required together with further studies to be performed in order to identify the key etiology and construct a clear guideline for management.

## Abbreviations

ANA: Antinuclear antibody
B-scan: Brightness scan
CCP: Cyclic citrullinated peptides
CT: Computed tomographyEEG Electroencephalogram
IOP: Intraocular pressure
OPG: Orthopantomogram
PRS: Parry–Romberg syndrome
VA: Visual acuity

## Authors’ information

**Dr Jimmy Girgis William Abdelnour**

MBBS, FCCS, MRCP (UK - Part 1), PGCert. DM (Queen Margaret University – Edinburgh).

**Dr Youeil Girgis William Abdelnour**

MBBS, MSc Pediatric Emergency (University of Edinburgh).

**Dr Rose-Mery Amin Boushra Kerollos**

MBBS.

**Dr Ziryab Imad Taha Mahmoud**

Consultant of Internal Medicine at Haj El-Safi Teaching Hospital,

Assistant Professor of Medicine and Head of Department of Internal Medicine at University of Bahri,

MBBS, MRCP (UK), MSc Clinical Pharmacology, Diploma of Research Methodology and Ethics, Rheumatology Fellowship trainee (SMSB).
